# Protein ubiquitination in T cell development

**DOI:** 10.3389/fimmu.2022.941962

**Published:** 2022-08-04

**Authors:** Ting Zhong, Kang Lei, Xiaoxi Lin, Zhiguo Xie, Shuoming Luo, Zhiguang Zhou, Bin Zhao, Xia Li

**Affiliations:** National Clinical Research Center for Metabolic Diseases, Key Laboratory of Diabetes Immunology, Ministry of Education, and Department of Metabolism and Endocrinology, The Second Xiangya Hospital of Central South University, Changsha, China

**Keywords:** T cell development, thymocyte, ubiquitination, E3 ubiquitin ligase, deubiquitinating enzyme

## Abstract

As an important form of posttranslational modification, protein ubiquitination regulates a wide variety of biological processes, including different aspects of T cell development and differentiation. During T cell development, thymic seeding progenitor cells (TSPs) in the thymus undergo multistep maturation programs and checkpoints, which are critical to build a functional and tolerant immune system. Currently, a tremendous amount of research has focused on the transcriptional regulation of thymocyte development. However, in the past few years, compelling evidence has revealed that the ubiquitination system also plays a crucial role in the regulation of thymocyte developmental programs. In this review, we summarize recent findings on the molecular mechanisms and cellular pathways that regulate thymocyte ubiquitination and discuss the roles of E3 ligases and deubiquitinating enzymes (DUBs) involved in these processes. Understanding how T cell development is regulated by ubiquitination and deubiquitination will not only enhance our understanding of cell fate determination *via* gene regulatory networks but also provide potential novel therapeutic strategies for treating autoimmune diseases and cancer.

## Introduction

Ubiquitin is a highly conserved protein of 76 amino acids and a versatile posttranslational modifier that is ubiquitously expressed in all eukaryotic cells (1). Protein ubiquitination plays a crucial role in protein homeostasis, thus regulating a vast array of biological processes, such as DNA damage and repair, cell cycle progression, apoptosis and cellular signaling (2, 3). Ubiquitin is added to the protein substrate *via* a subsequent enzymatic cascade by E1 ubiquitin-activating enzymes, E2 ubiquitin-conjugating enzymes and E3 ubiquitin ligases (4). The specificity of ubiquitination is mainly achieved by E3 ligases, which are responsible for substrate recognition *via* protein interacting domains and motifs (5). Ubiquitin has seven lysine residues that can be used to assemble polyubiquitin chains: Lys6, Lys11, Lys27, Lys29, Lys33, Lys48, and Lys63. A substrate can be polyubiquitylated or monoubiquitylated *via* polyubiquitin chains, and the impact of polyubiquitination on the target protein is greatly dependent on the type of conjugated chain (6). For example, except for Lys63, all six Lys linkages have been implicated in proteasomal degradation, with Lys48 and Lys11 being the predominant type of chains for substrate degradation in cells. Lys63-linked chains are involved in multiple nonproteolytic functions, including activation of NF-κB, DNA damage repair, and regulation of endosomal sorting pathways (7). Ubiquitination is a dynamic and reversible process, and ubiquitination induced by ubiquitin ligases can be counteracted by deubiquitinating enzymes (DUBs) to control the intensity and duration of ubiquitin signaling (8).

The thymus is the primary site for T cell development, thymic seeding progenitor cells (TSPs) arrive at the thymus from the bone marrow and initiate multistep maturation programs and checkpoints comprising lineage commitment, T cell receptor (TCR) gene rearrangement, and positive and negative selection. It is well established that thymocytes mature through ordered progression, including double-negative (CD4^–^CD8^–^, DN) stage, double-positive (DP) stage and CD4 or CD8 single-positive (SP) stages (9, 10). In the earlier DN1-3 stages, proliferation and differentiation are mainly driven by Notch signaling and cytokines such as c-kit and IL-7 (11). Then, cells successfully assembled pre-T cell receptor (pre-TCR) complexes will pass β-selection and transition from the DN3 to the DN4 stage. In DP stage, thymocytes undergo positive selection for self-human leukocyte antigen (HLA) recognition under the control of cortical thymic epithelial cells (cTECs) and negative selection to remove strong self-reactive clones based on the interaction with medullary thymic epithelial cells (mTECs) and thymic DCs (tDCs), finally becoming CD4^+^ SP or CD8^+^ SP cells (11). “Mature” SP thymocytes exit the thymus to the peripheral lymphoid organs (9).

**Figure 1:**
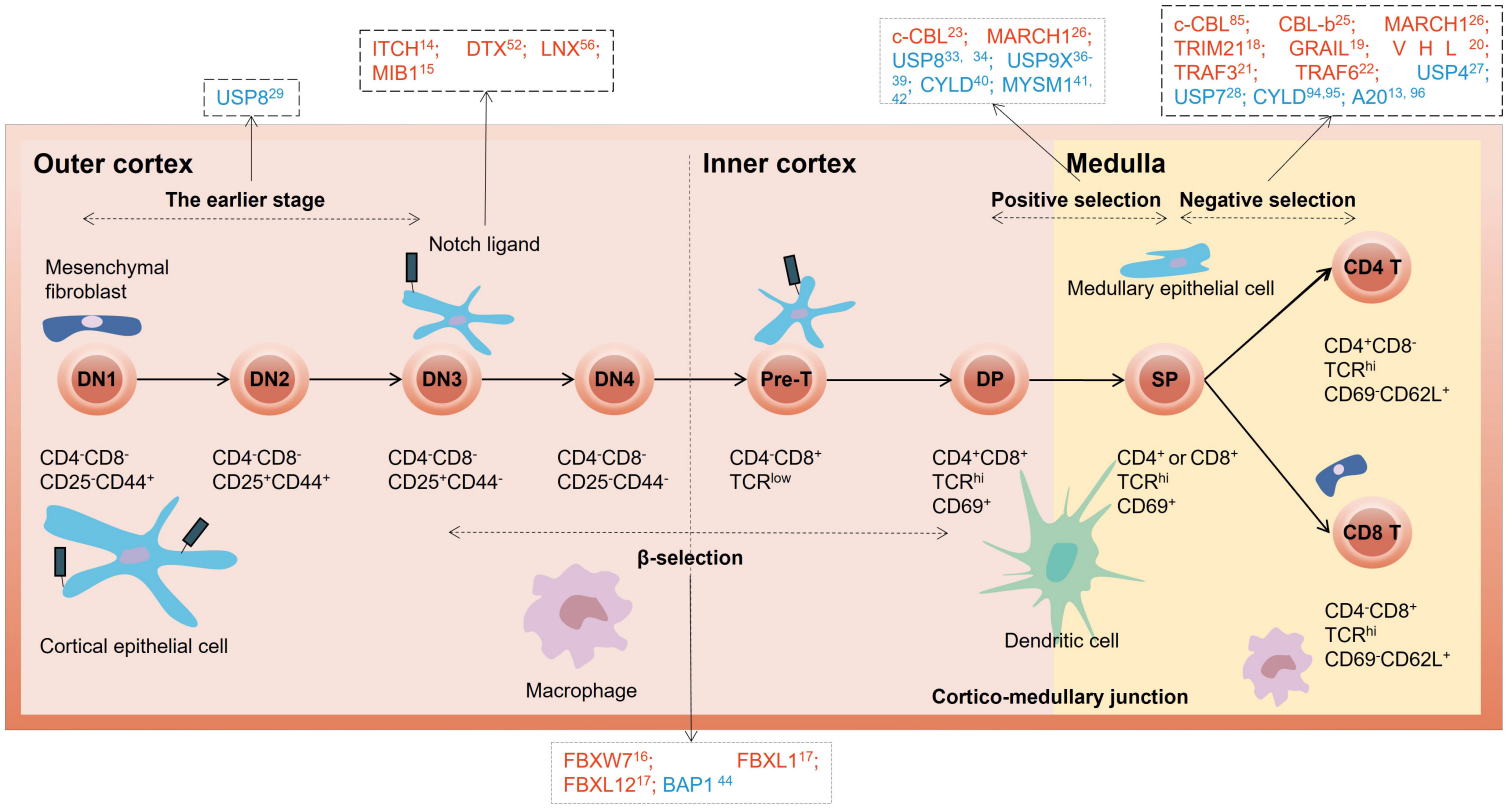
Overview of E3 ubiquitin ligases and DUBs in different stages of thymocyte development. The red letters in the black dotted box represent E3 ubiquitin ligases, and the blue letters represent DUBs. DN, double-negative; DP, double-positive; SP, single-positive.

Ubiquitin signaling modulates a variety of pathways involved in the T cell developmental process primarily through proteolysis-dependent mechanisms, such as Notch, pre-TCR signaling, Signal transducer and activator of transcription 3 (STAT3)-mediated signaling, Wnt signaling, and Nuclear factor κB (NF-κB) pathway (9, 12, 13). Here, we summarize the interplay between the ubiquitination system and T cell developmental programs (**Figure 1**). Specifically, we highlight the roles of E3 ligases and DUBs involved in these processes as well as the molecular mechanisms and cellular pathways that regulate thymocyte ubiquitination (**Table 1**).

**Table 1:** List of E3 ligases and DUBs that modulate T cell development.

Ubiquitinase	Substrate	Cko/ko mice	Phenotype	Ref.
**E3 ligases**				
Itch	Notch	Itch^-/-^; Lck-Notch1 tg^+^	-reduces DP and increases DN thymocytes -reduces apoptosis in the thymus and increases phospho-AKT signaling	(14)
Mib1	Dll1, Dll4	Mib1^-/-^	-impairs Dll1 and Dll4 endocytosis -reduces DP and increases DN thymocytes	(15)
Fbxw7	c-Myc	Lck-Cre; Fbxw7^fl/fl^	-promotes cell cycle exit -leads to hyperproliferation in thymocytes -increases DP thymocytes -enforces GATA3 expression	(16)
Fbxl1	Cdkn1b	Fbxl1^-/-^	-resultes in an incomplete DN3-DN4 developmental block	(17)
Fbxl12	Cdkn1b	Lck-Cre; Fbxl12^fl/fl^	-blocks DN3-DN4 transition	(17)
TRIM21	SOCS3	TRIM21^-/-^	-increases number of thymocytes -reduces frequency of DN cells	(18)
GRAIL	TCR-CD3	GRAIL^-/-^	-upregulates the function of tTregs	(19)
VHL	HIF-1α	Lck-Cre; Vhl^fl/fl^	-increases cell death and caspase activity -reduces TCR-mediated Ca^2+^ signaling	(20)
TRAF3	TCPTP	Lck-Cre; TRAF3^fl/fl^	-increases number of Treg cells in the thymus	(21)
TRAF6	NF-kB essential modifier (NEMO)	TRAF6^-/-^	-reduces autoimmunity -reduces Aire expression -reduces Treg cells	(22)
c-Cbl	CD5, TCRζ, Zap-70, SLAP, BIM	c-Cbl^-/-^	-increases TCR signaling -increases DP thymocytes -increased expression of CD3, CD5, and CD69 -enhances positive Selection of CD4^+^ T Cells	(23, 24)
Cbl-b	Foxp3, p85	Cbl-b^-/-^	-regulates tTregs -reduces mature SP thymocytes	(25)
MARCH1	MHCII	MARCH1^-/-^	-reduces tTregs	(26)
**DUBs**				
USP4	HUWE1	USP4^-/-^	-induces IR-induced apoptosis in thymus	(27)
USP7	Caspase 3	/	-regulates the apoptosis of thymocytes via interacting with caspase 3	(28)
USP8	GADS, CHMP5	CD4-cre;USP8^fl/fl^	-diminishes thymocyte proliferation	(29–34)
USP9X	Themis	USP9X^-/-^	-reduces thymic cellularity	(35–39)
CYLD	LCK	CYLD^-/-^	-regulates DP-SP transition	(40)
MYSM1	IRF2, IRF8	MYSM1^-/-^	-reduces thymus sizes and CD8^+^ T-cell numbers	(41, 42)
A20	GITR	CD4-cre;A20^fl/fl^	-increases CD69 expression within NKT thymocytes	(13, 43)
BAP1	H2AK119	Rosa26^CreERT2^; Bap1^fl/fl^	-causes a block at the DN3 stage	(44)

## E3 ubiquitin ligases in T cell development

E3 ligases are crucial components of the Ubiquitin Proteasome System. Several classes of these enzymes have been identified, known as the RING, U-box, HECT and RBR classes (45). As the last component of an enzymatic cascade, E3 ligases determine substrate specificity. Attaching ubiquitin to a protein could have profound effects on the protein’s cellular localization, protein-protein interactions or stability (46). Multiple E3 ligases have been demonstrated to play a role in T cell development.

### NOTCH-regulating E3 ligases mainly regulate the early stage of T cell development

Notch signaling has been identified as a key signaling pathway involved in the regulation of T cell development, especially in thymocyte survival, proliferation and differentiation (9, 47–49). E3 ubiquitin ligases that can catalyze the ubiquitylation of Notch include Itch, Ligand of Numb-Protein X (LNX), Deltex (DTX), Mind bomb (Mib) 1, Mib2, Neuralized (Neur) 1, and Neur2 (15). Itch binds to the N-terminal of the Notch intracellular domain *via* its WW domains and promotes ubiquitination of Notch *via* K29-linked ubiquitin chains, thus promoting its lysosomal degradation (50). Itch^-/-^ mice with an activated Notch1 transgene in their thymocytes show a reduction of DP and an increase of DN T cells, with a more severe autoimmune phenotype (14). Itch and Notch act in the AKT signaling concurrently in the genesis of autoimmune disease (14). In addition, Itch regulates Notch signaling *via* interacting with some molecules, such as Numb and DTX. Numb, an adapter protein, was initially identified as a negative regulator of Notch signaling. Numb binds to Itch WW domain and promotes ubiquitination and degradation of Notch1 by Itch (51). DTX, an E3 ligase, has been shown to be an itch homolog that plays a negative role in regulating Notch receptor signaling, and can cooperate with Itch to regulate NOTCH signaling *via* lysosomal degradation (52). In addition, downregulation of DTX in hematopoietic progenitors promotes T cell development in fetal thymic organ culture and *in vivo* (53). DTX antagonizes Notch1 signals by inhibiting coactivator recruitment (54) and restores DP thymocyte survival from the glucocorticoid (GC)-induced apoptosis by repressing SRG3 promoter activity (55). LNX can also cause proteasome-dependent degradation of Numb and therefore enhance Notch signaling (56). Mib1 modulates Notch signaling by ubiquitinating the Notch receptors (Dll1 and 4), promoting their endocytosis (57). Reciprocal bone marrow (BM) transplantation experiments revealed that Notch signaling was diminished in the DN thymocytes of Mib1 conditional KO mice (15). Furthermore, knocking down Mib1 in the coculture system causes a delay in T cell growth and a failure of Dll1 endocytosis (15).

### SCF complexes play crucial roles in thymic β-selection mediated cell proliferation

The SCF (Skp1-cullin-F-box protein) complex is a well-described multisubunit RING-finger E3 composed of Skp1, Cdc53/cullin, and an F box protein (58). Fbxw7 (F-box and WD-40 domain protein 7)—also known as Fbw7—is an SCF ubiquitin ligase component reported to play a role in thymocyte cell cycle progression by controlling the degradation of c-Myc, c-Jun, cyclin E, and Notch (59). Fbw7 modulates cell cycle progression by controlling c-Myc protein stability, and loss of Fbxw7 leads to hyperproliferation of thymocytes (16). Moreover, the SCF subunits Fbxl1 and Fbxl12, which are transcriptionally induced by Notch and pre-TCR signaling respectively, function identically but additively to promote the degradation of Cdkn1b and proliferation of β-selected thymocytes (17, 60). Deletion of Fbxl1 or Fbxl12 results in an incomplete DN3-DN4 developmental block and a reduced thymus size (17).

### TRIM family proteins have crucial roles during negative selection

As RING-type E3 ligases, tripartite motif (TRIM) proteins have been demonstrated to regulate the innate immune response (61, 62). However, recent studies suggest that TRIM21 alters T cell development in the thymus (63). TRIM21^−/−^ mice had an increased number of thymocytes and a reduced frequency of DN cells (18). TRIM21 targets suppressor of cytokine signaling-3 (SOCS3) for proteasomal degradation, thus impairing STAT3 activation in TECs (64). STAT3-mediated signaling has been shown to promote quintessential growth of mTECs (but not cTECs) (12, 65). Double-positive (DP) cells are selected by cTECs to become CD4 or CD8 SP cells (66), while SP thymocytes are further negatively selected in the medulla (67). We can surmise that TRIM21 plays a crucial role during negative selection in the thymus.

### GRAIL and VHL regulate T cell development during negative selection

Gene related to anergy in lymphocytes (GRAIL) is a RING-type E3 ligase required for the initiation of CD4^+^ T cell anergy *in vivo*. Previous studies considered GRAIL expression patterns in murine CD4^+^ T cells as a defined anergic phenotype and a negative regulator of the immune response (68, 69). Notably, GRAIL expression is upregulated in tTregs, and its overexpression in DO11.10 T cells convert these cells to a regulatory phenotype (19). Nurieva et al. reported that GRAIL regulates Treg cell function by mediating TCR-CD3 degradation (70). Works are needed to delineate the mechanism(s) of how GRAIL mediates its suppressor activity in the thymus. The von Hippel-Lindau (VHL) is a RING-type E3 ligase that targets hypoxia-inducible factor-1α (HIF-1α) for proteasomal degradation (20). Vhl-deficient mice had a severe reduction in thymus sizes and thymic cellularity due to enhanced caspase 8 activity in the apoptotic pathway, as a result of HIF-1α accumulation (20).

### TRAF family proteins regulate T cell development during negative selection

Tumor necrosis factor receptor (TNFR)-associated factor 3 (TRAF3) is a member of the TRAF family of cytoplasmic adaptor proteins and plays a role in modulating IL-2 signaling in T cells. T cell conditional TRAF3 knockout mice resulted in an increased number of Treg cells in the thymus (21) due to more efficient conversion of CD25^+^ Foxp3^–^ Treg precursors to CD25^+^ Foxp3^+^ mature Treg cells (71). TRAF6 is another adaptor E3 ligase that is involved in central tolerance by regulating the development of thymic stroma. TRAF6^−/−^ fetal thymic stroma tissue fails to mediate negative selection (22). Furthermore, specific deletion of TRAF6 in TECs hinders the growth of mTECs (72). Several studies have suggested that TRAF6 regulates the establishment of thymic microenvironments through manipulating RelB (73), RANK (74) and CD40 (75) expression.

### Cbl family proteins regulate multiple stages of T cell developmental processes

The Casitas B-lineage lymphoma (Cbl) family of proteins are RING-finger domain containing E3 ubiquitin ligases (76, 77). In mammals, two highly homologous adaptor proteins of the Cbl family, c-Cbl and Cbl-b, are involved in the negative regulation of the immune system (78, 79). Both c-Cbl and Cbl-b contain a highly conserved amino-terminal tyrosine-kinase binding (TKB) domain, a less conserved carboxyl-terminal proline-rich region (PRR) and a RING finger. Through their protein-protein interaction domains, c-Cbl and Cbl-b form multiple complexes together with several signaling molecules to regulate intracellular signaling events (80). The first evidence indicating that Cbl proteins are associated with thymic selection came from experiments showing that thymocytes from c-Cbl^-/-^ mice have increased signaling through the TCR and CD4^+^ CD8^+^ DP thymocytes exhibited increased expression of CD3, CD5, and CD69 in the c-Cbl knockout (KO) model (23). Moreover, c-Cbl selectively inhibits thymic-positive selection of CD4 but not CD8 T cells (23). This suggests that the positive selection of thymocytes bearing MHC class II-restricted TCRs is negatively regulated by c-Cbl. Mechanistically, c-Cbl modulates CD4^+^ T-cell development by promoting TCR-ζ lysosomal degradation. In this model, a transient trimolecular complex of TCRζ-Zap-70-Cbl is formed, and ubiquitin is then shifted from the Cbl-E2 complex to TCRζ (79, 81). In addition to Zap-70, Src-like adaptor protein (SLAP) might also act as a bridge to bond TCRζ and Cbl. In support of this, SLAP^-/-^ mice were shown to have a similar phenotype to c-Cbl^-/-^ mice (82–84). In addition to positive selection, c-Cbl also regulates thymocyte negative selection, probably by ubiquitinating and proteasomal degrading the pro-apoptotic molecule B-cell lymphoma 2-interacting mediator of cell death (BIM) (85). Furthermore, deactivation of c-Cbl reverses T cell developmental detention in SLP-76-deficient mice, in which T cell development is impeded at the DN3 stage (24). In conclusion, the c-Cbl protein modulates multiple stages of T cell developmental processes.

Analyses of Cbl-b KO mice resulted in no similar findings (86). Given that the expression level of Cbl-b in thymocytes is much lower than that of c-Cbl, it would not be surprising. However, Zhao Y et al. reported that Cbl-b, together with Stub1, regulates thymic-derived CD4^+^ CD25^+^ regulatory T cells (tTregs) development by targeting Foxp3 for ubiquitination and degradation in the proteasome (25). Moreover, Raberger J et al. reported that the CD4/CD8 developmental profile was noticeably altered and mature SP thymocytes were absent in Vav1^-/-^ or ITK^-/-^ thymocytes (87), and the signaling defects in Vav1^-/-^ or ITK^-/-^ thymocytes can be rescued upon deletion of Cbl-b (87). These results indicate that Cbl-b alters thymus development.

### MARCH family E3 ligases modulate the development of tTregs

Membrane-associated RING-CH1 (MARCH1) is an E3 ubiquitin ligase that regulates MHCII ubiquitination (26). Thymocytes and TECs scarcely express MARCH1, while DCs in the thymus express comparatively high levels of MARCH1 (26). MARCH1 deficiency results in an elevated level of MHCII, which leads to a considerable decline in the number of thymic Treg (tTreg) cells but not conventional CD4^+^ T cells in mice (26). Another E3 ligase, MARCH8, is responsible for MHC II ubiquitination specifically in thymic epithelial cells. In MARCH8^-/-^ mice, TECs express elevated levels of MHC II, but the development of conventional CD4^+^ T cells or tTreg cells remains unchanged. It is possible that tTreg development does not require MHC II ubiquitination in TECs (88).

## DUBs in T cell development

In addition to E3 ligases, the ubiquitin system is also regulated by DUBs. Ubiquitin chains can be removed from the substrate by DUBs, which are essential for the dynamic regulation of the protein ubiquitination process (89, 90). Several DUBs have been identified as regulators in the T cell developmental program.

### USP family proteases regulate multiple stages of T cell developmental processes

Ubiquitin-specific proteases (USPs) are the largest subfamily of DUBs and contain more than 100 members (91). Ubiquitin-specific peptidase 4 (USP4) has been shown to inhibit p53 signaling through interacting with and stabilizing ARF-binding protein 1 (ARF-BP1, also known as HUWE1), an E3 ligase for p53 (24). USP4 knockout mice are viable and fertile but exhibit enhanced ionizing radiation (IR)-induced thymocyte apoptosis (27). In addition, USP4, a DUB with dual hydrolyzing activity for K48- and K63-conjugated polyubiquitin chains, interacts with the Nemo like kinase (Nlk) and T-cell factor (TCF) 4, two known components of the Wnt pathway that are essential for cell development (92). USP7 (also known as HAUSP), which is highly expressed in the thymus, also regulates the apoptosis of thymocytes during negative selection *via* caspase-dependent signaling (28). Likewise, the processing of HAUSP does not occur in caspase 3-deficient thymocytes (28). Ubiquitin-specific protease USP8 is a deubiquitinase involved in the endosomal sorting complex required for transport (ESCRT) system (93). A recent study reported that USP8 is involved in thymocyte maturation and proliferation processes by modulating the Foxo1-IL-7Rα axis (29). Moreover, the amino-terminal SH3BM of USP8 binds with higher affinity to the TCR adaptor GADS in a caspase-dependent manner (30–32). Another study identified USP8 as a deubiquitinase for CHMP5, a component of the ESCRT complex, and uncovered the role of the CHMP5-USP8 complex in regulating thymic positive selection (33, 34). Ubiquitin-specific protease 9X (USP9X) is a member of the peptidase C19 family and encodes a protein similar in structure to ubiquitin-specific proteases. Deletion of Usp9X resulted in an overall reduction in thymic cellularity (35). Mechanistically, USP9X interacts with and stabilizes Themis, an important TCR signaling protein (36), by removing ubiquitin K48-linked chains on Themis upon TCR stimulation, thus affecting thymic positive selection (37–39).

### CYLD regulates T cell development during negative selection

Cylindromatosis (CYLD) is a lysine 63-deubiquitinating enzyme that positively regulates TCR signaling by promoting the recruitment of Lck to its substrate, Zap70, in thymocytes (40). CYLD-deficient mice displayed significantly fewer mature CD4^+^ and CD8^+^ single-positive thymocytes (40). Previous studies identified CYLD as a switch in T cell development during the transition from double-positive to single-positive thymocytes (40). Furthermore, S. Reissig et al. demonstrated impaired negative selection in the thymus of CYLD^ex7/8^ mice, which overexpresses the naturally occurring CYLD splice variant short CYLD (sCYLD), whereas full-length CYLD (FL-CYLD) is absent (94, 95).

### MYSM1, A20 and BAP1 modulate multiple stages of T cell developmental processes

Other types of DUBs involved in T cell development include Myb-like SWIRM and MPN domain containing 1 (MYSM1), A20 and BRCA1-associated protein-1 (BAP1). Conditional ablation of histone H2A deubiquitinase MYSM1 at late stages of thymic development in a mouse model showed a severe reduction in thymus sizes and CD8^+^ T-cell numbers, indicating the critical role of MYSM1 in the positive selection of CD8^+^ T cells (41, 42). A20, also known as TNF-α-induced protein 3 (TNFAIP3), regulates tTreg development and maturation by restraining the activation of NF-kB signaling (13, 96). T lineage cell conditional A20 knockout mice showed that tTreg cell compartments are quantitatively enlarged (13). In addition, A20 specifically limits TCR-dependent activation of NKT cells in the thymus (43). BAP1 is a member of the ubiquitin C-terminal hydrolase (UCH) subfamily of DUBs and has been shown to be involved in β-selection mediated cell expansion (44). BAP1 deletion in adult mice led to serious thymic atrophy and loss of cellularity due to defects in cell proliferation (97). Likewise, BAP1 deficiency caused a block at the DN3 stage before the pre-TCR checkpoint by facilitating the ubiquitination of histone H2A at Lys^119^ (H2AK119) (97).

## Conclusion

During the past few years, several lines of evidence have shown that T cell development is regulated at multiple levels; in addition to transcriptional control, posttranslational regulation also plays a crucial role in those processes (9, 98, 99). An increasing number of studies using transgenic mouse models have demonstrated that E3 ubiquitin ligases and DUBs are involved in specific stages of thymocyte maturation by modulating the activity or stability of key proteins during cellular signal transduction cascades (98, 99). Technological advancements in single-cell proteomics, CRISPR/Cas9 mutagenesis and mass cytometry will continue adding valuable findings to this area of research. Future work on the molecular mechanisms of ubiquitination and deubiquitination in T cells will not only enhance our understanding of cell fate determination *via* gene regulatory networks but also provide potential novel therapeutic strategies for treating autoimmune diseases and cancer.

## Author contributions

XLi and BZ: conceptualization and guidance. TZ: writing the original draft. KL: visualization. XLn: provide assistances. ZZ and SL: proofreading. All authors contributed to the article and approved the submitted version.

## Funding

This work was supported by the Natural Science Foundation of China (Grant NO. 82170795, 8207034059) and the Science and Technology Innovation Program of Hunan Province (Grant NO. 2020RC4044).

## Conflict of interest

The authors declare that the research was conducted in the absence of any commercial or financial relationships that could be construed as a potential conflict of interest.

## Publisher’s note

All claims expressed in this article are solely those of the authors and do not necessarily represent those of their affiliated organizations, or those of the publisher, the editors and the reviewers. Any product that may be evaluated in this article, or claim that may be made by its manufacturer, is not guaranteed or endorsed by the publisher.
